# Comparison of the performance of magnetic targeting drug carriers prepared using two synthesis methods†

**DOI:** 10.1039/d1ra04256d

**Published:** 2021-06-09

**Authors:** Zhen Shi, Yazhen Wang, Shaobo Dong, Tianyu Lan

**Affiliations:** College of Materials Science and Engineering, Qiqihar University Qiqihar 161006 Heilongjiang China wyz6166@qqhru.edu.cn; Heilongjiang Provincial Key Laboratory of Polymeric Composite Materials Qiqihar 161006 China; College of Chemistry, Chemical Engineering and Resource Utilization, Northeast Forestry University Harbin 150040 Heilongjiang China

## Abstract

In this paper, two methods were used to prepare the magnetic targeting drug carrier Fe_3_O_4_–PVA@SH, the step-by-step method and the one-pot method. The loading and release properties of the compound were measured. The results show that the Fe_3_O_4_–PVA@SH prepared using both methods exhibited excellent drug delivery properties in an environment that simulates human body fluid (pH 7.2) and a lysosomal *in vitro* simulation (pH 4.7). In applications such as drug delivery, magnetic targeted drug carriers prepared by both methods demonstrated superparamagnetism, high fat solubility, high hydroxyl content, and good water solubility.

## Abstract 2

Roadmap for the synthesis of Fe_3_O_4_–PVA@SH using the step-by-step method and one-pot method.

## Graphical abstract

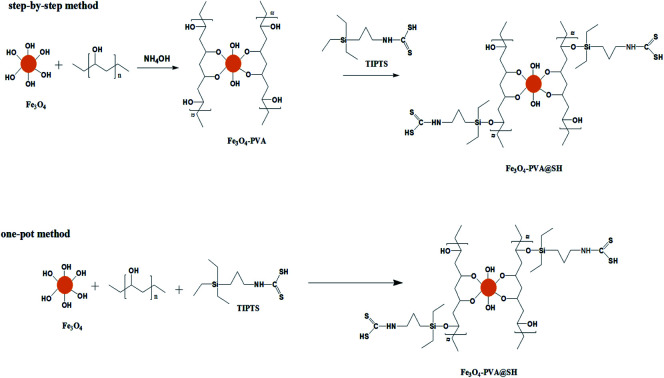

## 1. Introduction

As more interdisciplinary work is conducted in the fields of nanotechnology and pharmacology, magnetically targeted drug delivery systems have attracted increasing interest. These delivery systems are specifically designed to overcome the shortcomings associated with traditional drug delivery mechanisms, including poor biodistribution, high toxicity, and poor sensitivity.^1,2^ By using modified magnetic particles as drug carriers and enriching magnetic drug particles in the lesion, the loaded drug is released in a controlled manner to achieve targeted therapy. Thus, the magnetically targeted drug delivery system achieves the four-fold objective of synergism, toxicity reduction, controlled release, and gradual release.^3,4^

New opportunities have become available in general and applied research on biomedical diagnostics and therapeutics through the recent development of small-size, high-saturation magnetization strength, superparamagnetic, and surface-modified magnetic nanoparticles.^5–7^ The size of these magnetic nanoparticles are comparable to viruses (20–450 nm), proteins (5–50 nm), DNA, and genes (2 nm and 10–100 nm long).^8,9^ Therefore, when used as magnetic targeting drug carriers, these nanoparticles are capable of entering the target site of a diseased organ or tissue as well as the interior of tumour cells.^10–13^ For example, by loading adriamycin (DOX) into hollow spikelets and encapsulating these hedgehog-shaped objects with photothermally fusible gelatin, Wang Jie and Zhou Jiahong *et al.* achieved a controlled surface morphological transition from quasi-spherical to spiky. The transition was facilitated by the intense photothermal action of FeSe_2_ and the release of DOX, which leads to synergistic tumour suppression and immunogenic tumour cell death.^14^ Mallika Modak's team used rapid nanoprecipitation techniques to co-load a drug, embedding molecules with different chemical properties in BCN (MBCN) nanoparticles to produce the copolymer PEG-*b*-PPS BCN. This copolymer is characterised by a unique and highly organised cubic-phase nanostructure after intravenous administration. *In vivo* biodistribution analysis demonstrates universal encapsulation and delivery capabilities for hydrophilic and hydrophobic payloads.^15^

The carbohydrate polymer PVA is characterized by its water solubility, film-forming ability, adhesion capability, ability to form emulsions, and excellent resistance to oil and solvents. PVA has found wide use in pharmaceutical carriers because it is non-toxic, odourless, non-irritating to the skin, unlikely to trigger skin allergies, elastic, and is known for its good biological adaptability.^16–20^ Sulfhydryl groups are also widely applied to drug carrier development to improve fat solubility. Notably, Qian Zhang prepared cysteamine (CS)–gold nanoparticles (AuNPs)–adriamycin (DOX-SH) for fluorescence-enhanced cell imaging and targeted drug delivery.^21^

**Scheme 1:**
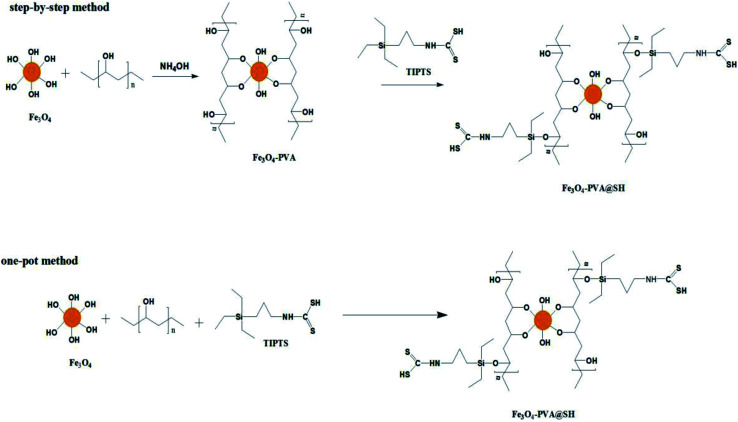
Roadmap for the synthesis of Fe_3_O_4_–PVA@SH using step-by-step method and one-pot method.

In this paper, a new magnetic targeting drug carrier (Fe_3_O_4_–PVA@SH) was prepared using the step-by-step method and the one-pot method (Scheme 1). Both methods allow for the creation of Fe_3_O_4_–PVA@SH drug carriers with uniform dimensions and excellent superparamagnetic properties. The sulfhydryl groups (–SH), amino groups (–NH) and hydroxyl groups (–OH) of the drug carrier and the polar groups [carboxyl groups (–COOH), hydroxyl groups (–OH)] are all capable of forming stable hydrogen bonds on the drug carrier, which allows for the stabilisation of the drug load. In this study, aspirin and adriamycin hydrochloride (DOX·HCl) were used as model drugs to measure the loading capacity and drug release properties of Fe_3_O_4_–PVA@SH.

## 2. Experiments

### 2.1 Materials

Polyvinyl alcohol (PVA, *M*_w_ 89 000 Da, Aladdin Reagents (Shanghai) Co.), ammonia (AR, Tianjin Kaitong Chemical Reagent Co., Ltd., China) dimethyl sulfoxide (DMSO, AR, Sinopharm Holdings Chemical Reagent Co., Ltd.), aspirin (acetylsalicylic acid, aspirin, 99%, purchased from Sinopharm Holdings Chemical Reagent Co.). Adriamycin hydrochloride (DOX·HCl, 99%) was purchased from Sinopharm Holding Chemical Reagent Co. Fe_3_O_4_ was manufactured in-house (particle size of 65.17 nm). TIPTS was manufactured in-house.^22^

### 2.2 Step-by-step method for preparing Fe_3_O_4_–PVA@SH^23^

#### 2.2.1 Synthesis of Fe_3_O_4_–PVA

First, an appropriate amount of PVA was dissolved in 100 mL of distilled water and dissolved using a mechanical stirrer. The Fe_3_O_4_ was sonicated for one hour and then added to the PVA solution. An ammonia solution was then added to adjust the pH to the desired value. This reaction was carried out under nitrogen protection for 5 hours. The resulting black precipitate was washed with distilled water until a neutral pH was achieved.

#### 2.2.2 Fe_3_O_4_–PVA@SH synthesis

Appropriate amounts of TIPTS and Fe_3_O_4_–PVA were dispersed into 100 mL of DMSO. After 2 hours of sonication, H_2_SO_4_ was added until the pH of the solution system was 1–2. The product was then filtered, rinsed with water, and freeze dried until used.

### 2.3 Preparation of Fe_3_O_4_–PVA@SH by the one-pot method

The appropriate amount of Fe_3_O_4_, PVA and TIPTS were mixed into an aqueous DMSO solution (25 : 1). The appropriate amount of ammonia (NH_3_·H_2_O) was then added in a nitrogen-protected environment, and stirred for 5 h at 20 °C, which produced a black solid. The black solid was repeatedly washed with distilled water to obtain the final product, and freeze-dried until used. Some of this product's most important features are listed in the following sub-sections.

### 2.4 Characterisation

Fourier transform infrared (FTIR) light testing was performed with an IRAffinity-1 spectrometer. Scanning Electron Microscopy (SEM) and Energy Dispersive Spectroscopy (EDS) images were recorded using a JSM-6380 LV microscope.

### 2.5 Measurement of the swelling rate

Eqn (1) was used to calculate the swelling rate and analyse the swelling properties of Fe_3_O_4_–PVA@SH prepared using the step-by-step and one-pot methods.1
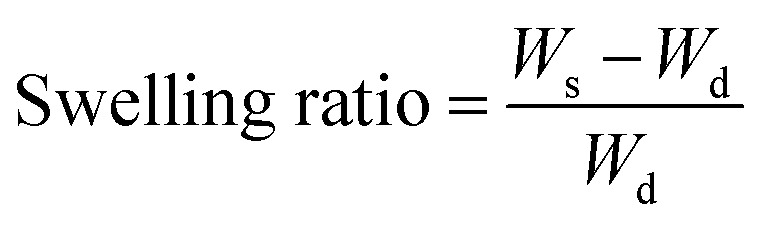
*W*_d_ and *W*_s_ are the weights of dried Fe_3_O_4_–PVA@SH before and after immersion in an aqueous solution for 48 hours, respectively.

### 2.6 Drug carrying capacity

We placed an appropriate concentration of Fe_3_O_4_–PVA@SH, which had been prepared using one of the two methods, in an aqueous aspirin/DOX·HCl solution at 37 °C. This was done to analyse the drug carrying capacity of Fe_3_O_4_–PVA@SH prepared using both methods. The supernatant was then analysed using a UV spectrophotometer after a predetermined time interval. The Fe_3_O_4_–PVA@SH loadings were calculated with the following equation:2
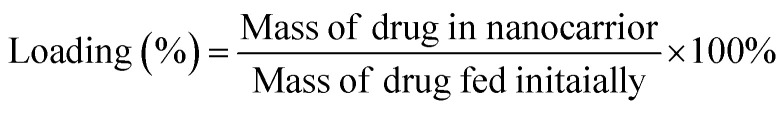


### 2.7 Drug release

To determine the drug release from Fe_3_O_4_–PVA@SH prepared using each method of preparation, a UV-visible spectrophotometer was used to measure concentration change over time. The two drug-loaded Fe_3_O_4_–PVA@SH compounds were placed in dialysis bags in PBS at 37 °C with pH levels of 4.7 and 7.2, respectively. The release of the drug within the body was simulated by an oscillograph set to a certain vibration frequency. At specific time intervals, the supernatant was collected for analysis using a UV spectrophotometer. Each experiment was repeated three times. The following equation was used to calculate the amount of drug released from the Fe_3_O_4_–PVA@SH:3
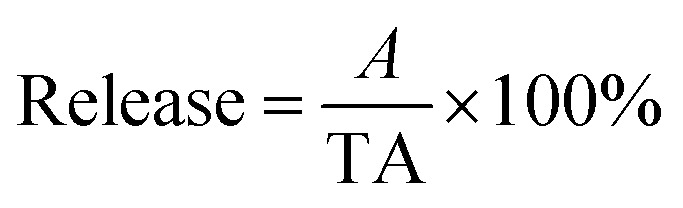
*A* is the drug release from Fe_3_O_4_–PVA@SH at time *t*, and TA is the total release from Fe_3_O_4_–PVA@SH.

## 3. Results and discussion

### 3.1 FT-IR analysis

**Fig. 1:**
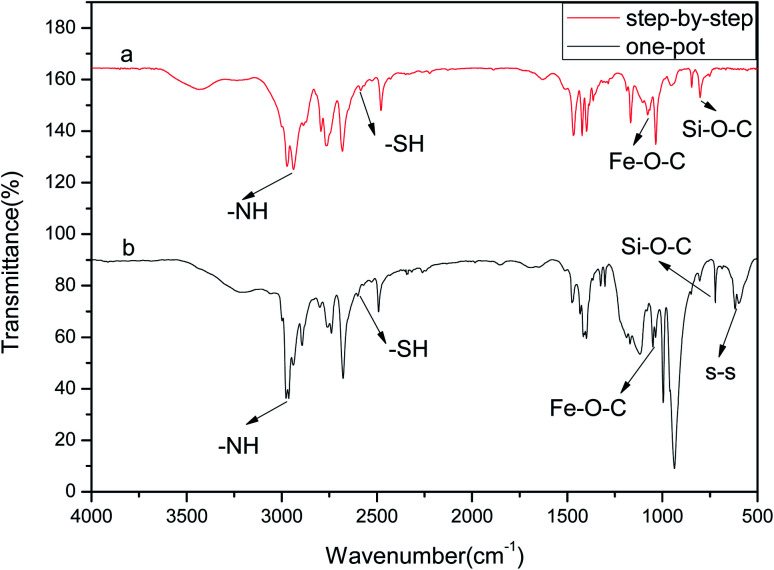
FTIR of the drug carrier Fe_3_O_4_–PVA@SH prepared using the step-by-step method (a) and the one-pot method (b).

The FTIR analysis of the drug carrier Fe_3_O_4_–PVA@SH prepared using each of the two methods can be seen in Fig. 1(a). The peak at 2583 cm^−1^ in Fig. 1(a) is the –SH. The height of the peak at 1432 cm^−1^ is caused by the hydrocarbon bending vibration of –CH_3_, and the stretching vibration of –CH causes the peak at 2928 cm^−1^. The height of the peak at 1373 cm^−1^ is the stretching vibration of –C–C–. The height of the peak at 1083 cm^−1^ represents the stretching vibration peak of Fe–O–C.^24^ The height of the peak at 1628 cm^−1^ is the stretching vibration peak –NH, while the peak at 798 cm^−1^ can be attributed to the stretching vibration peak of Si–O–CH_3_.

Fig. 1(b) shows the infrared spectra of Fe_3_O_4_–PVA@SH produced by the one-pot method (b). The presence of Fe–O–C and –SH indicates the successful synthesis of Fe_3_O_4_. This was encapsulated to form the magnetic core drug carrier system, Fe_3_O_4_–PVA@SH, which contains thiols and hydroxyl groups. The peak at 2573 cm^−1^ in Fe_3_O_4_–PVA@SH represents the performance of –SH. The height of the peak at 1414 cm^−1^ is caused by the hydrocarbon bending vibration of –CH_3_, while the peak at 2933 cm^−1^ indicates the stretching vibration of –CH. The stretching vibration of –C–C– caused the height of the peak at 1392 cm^−1^. The peaks at 1041 cm^−1^ and 1647 cm^−1^ are the stretching vibration peaks of Fe–O–C and –NH, respectively. The ridge at 797 cm^−1^ can be attributed to the stretching vibration peak of Si–O–CH_2_. The irregular motion collision of molecules in the experimental system produced the telescopic vibration peak at 580 cm^−1^, where some sulfhydryl groups formed disulfide bonds (–S–S–).

A difference occurred using the step-by-step method and one-pot method, a partial chemical shift of the functional groups of the prepared Fe_3_O_4_–PVA@SH, which we believe was due to a difference in the first step of each method. The first step of the step-by-step method of Fe_3_O_4_–PVA preparation results in Fe_3_O_4_ being encapsulated in PVA, whereas the one-pot preparation allows for greater amounts of space and thus further opportunity for Fe_3_O_4_, TIPTS and PVA to encounter each other. In the latter method, more covalent bonds form between these three molecules, resulting in an uneven distribution of the electron cloud between the chemical bonds, and thus partial chemical shifts. However, the presence of Fe–O–C and –SH in the FTIR spectra of both the step-by-step (Fig. 1(a)) and the one-pot method (Fig. 1(b)) indicates the successful synthesis of Fe_3_O_4_ that is wrapped to form a new magnetic amphiphilic drug carrier Fe_3_O_4_–PVA@SH with thiols and hydroxyl groups.

### 3.2 XRD analysis

**Fig. 2:**
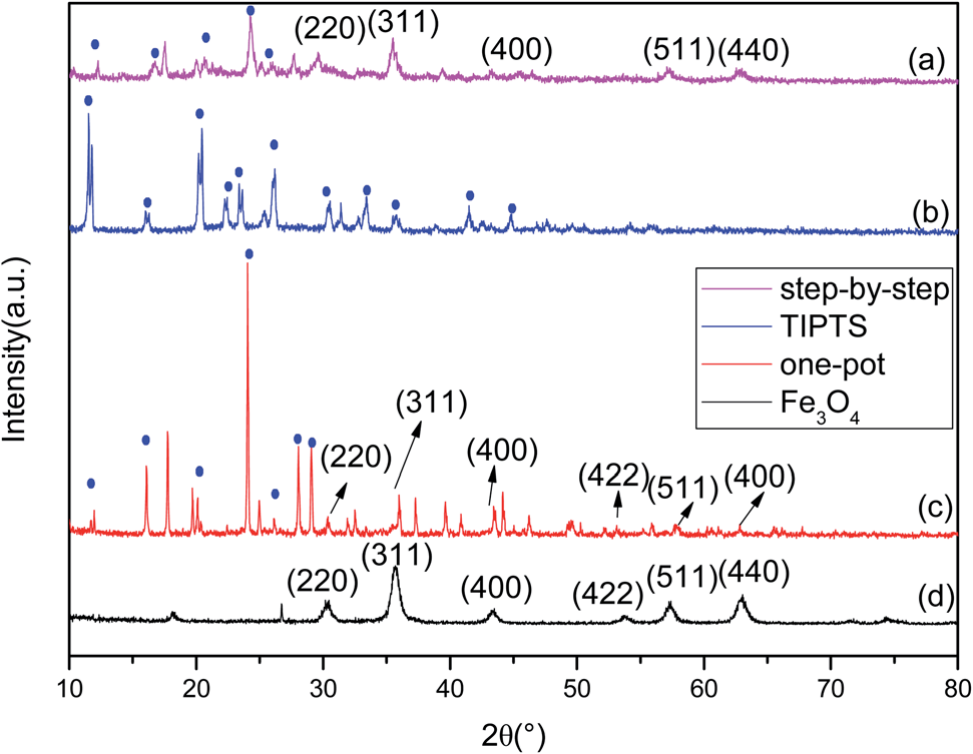
XRD of the drug carrier Fe_3_O_4_–PVA@SH prepared using the step-by-step method (a), TIPTS (b), the one-pot method (c), and Fe_3_O_4_ (d).

Fig. 2(d) shows the XRD pattern of Fe_3_O_4_. According to the standards card for Fe_3_O_4_ (JCPDS card no. 72-2303),^24–26^ the peaks associated with Fe_3_O_4_ are on the following planes (220), (311), (400), (422), (511) and (440), which correspond to peaks at 2*θ* = 30.1°, 35.5°, 43.3°, 54.21°, 57.3° and 62.7°. Fig. 2(b) shows the peak pattern of TIPTS, with peaks at 2*θ* = 11.7°, 16.4°, 20.11°, 22.14°, 23.56°, 26.07°, 30.37°, 33.47°, 35.62°, 41.7°, and 44.69°. We compared the XRD patterns of the drug carrier Fe_3_O_4_–PVA@SH prepared using the step-by-step method, shown in Fig. 2(a), and the one-pot method, shown in Fig. 2(c). The step-by-step method for the synthesis of the drug carrier shows that the typical crystalline forms of the Fe_3_O_4_ are (220), (311), (400), (511) and (440). The absence of the characteristic peak of (422) is because in the step-by-step method, the outside of the Fe_3_O_4_ is covered by PVA, and PVA has a partially masking modification effect on the Fe_3_O_4_ within. The typical crystalline shape of the TIPTS can also be seen in the step-by-step method. Due to the bent and folded structure of the carbon backbone of the PVA, the PVA has a masking modification effect on some of the crystalline shapes in the outermost TIPTS, making some of the crystalline shapes of the TIPTS less visible when using the step-by-step method. The typical crystalline forms of Fe_3_O_4_ [(220), (311), (400), (422), (511), (440)] can also be seen in the drug carriers prepared by the one-pot method. However, since Fe_3_O_4_, PVA and TIPTS are added at the same time in the one-pot method, there is equal time and opportunity for contact with the other raw material, resulting in a difference in the crystal shapes obtained using each methods.

### 3.3 SEM and particle size analysis

**Fig. 3:**
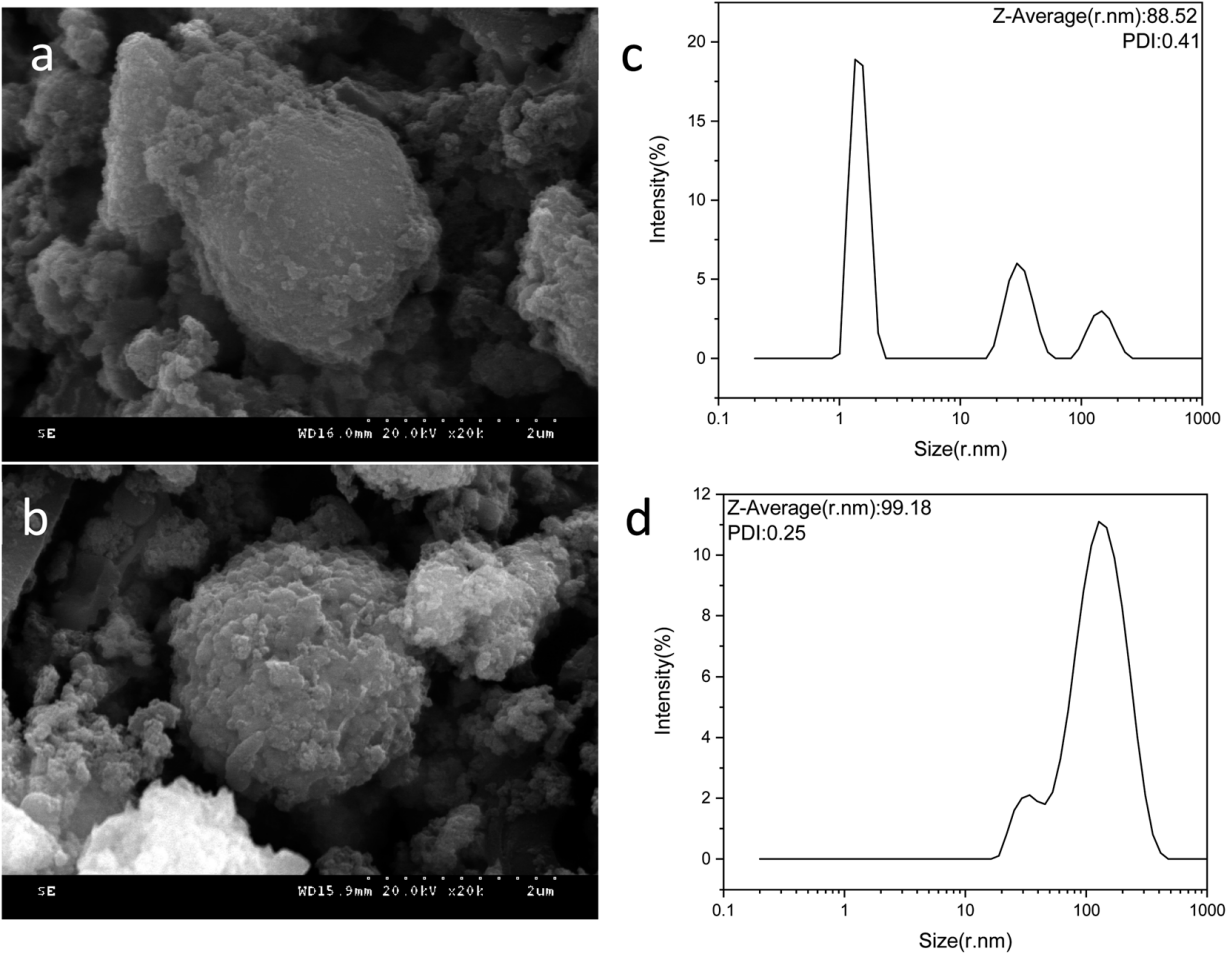
SEM image of the step-by-step method Fe_3_O_4_–PVA@SH (a) and one-pot method product (b). Particle size distribution of the step-by-step method Fe_3_O_4_–PVA@SH (c) and one-pot method (d).

Fig. 3(a) and (b) show SEM images of Fe_3_O_4_–PVA@SH nanoparticles prepared using each of the two methods. The spheroid is Fe_3_O_4_ and the outermost irregularity of the spheroid is a mixture of PVA and TIPTS.

Fig. 3(c) shows the particle size distribution of the Fe_3_O_4_–PVA@SH prepared using the step-by-step method. The average particle size is 88.52 nm and PDI = 0.41. We can see from the particle size distribution that the step-by-step prepared drug carriers are distributed in the range of 1–10 nm, 10–100 nm, and 100–1000 nm. 1–10 nm represent mostly single dispersed Fe_3_O_4_ nanoparticles, and 10–100 nm are the step-by-step prepared Fe_3_O_4_–PVA@SH compounds with uniform size dispersion. 10–1000 nm particles occur in the agglomeration of Fe_3_O_4_–PVA@SH prepared using the step-by-step method.

Fig. 3(d) shows that the average particle size of the Fe_3_O_4_–PVA@SH nanoparticles prepared by the one-pot method is 99.19 nm, PDI = 0.25. We can see that there are two distribution intervals for the particle size, 10–100 nm and 100–1000 nm. We hypothesize that the distribution interval of 100–1000 nm is due to the one-pot process, which causes some of the nanoparticles to agglomerate. The particle size distribution plots of Fe_3_O_4_–PVA@SH prepared using the step-by-step and one-pot methods, shown in Fig. 3(c) and (d), show that the particle size from the one-pot method is larger than from the step-by-step method. In the distribution process, PVA is first grafted with Fe_3_O_4_. PVA also acts as a dispersant, which allows Fe_3_O_4_ to be evenly distributed. However, during the preparation using the one-pot method, Fe_3_O_4_ is simultaneously in contact with PVA and TIPTS. In addition, Fe_3_O_4_ and TIPTS compete for the –OH binding site of PVA. Therefore, it is evident that the dispersive effect of PVA is not optimal, resulting in a larger particle size using the one-pot method than with the step-by-step method.

### 3.4 EDS analysis

**Fig. 4:**
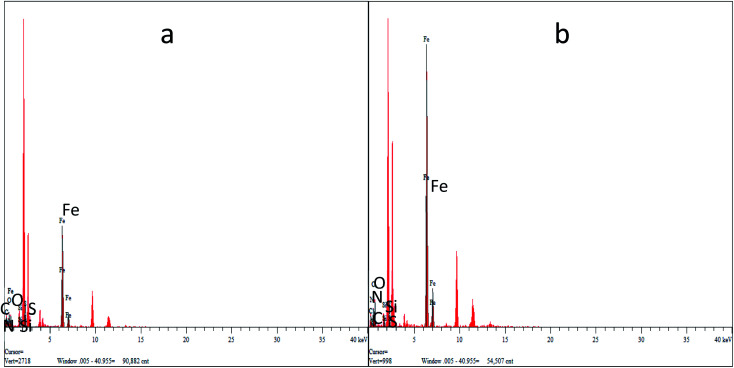
EDS for Fe_3_O_4_–PVA@SH prepared using the step-by-step method (a) and the one-pot method (b).

Fig. 4(a) and (b) show the EDS energy distribution of Fe_3_O_4_–PVA@SH produced by the two methods. We carried out a spot scan of the Fe_3_O_4_–PVA@SH and determined that the elements C, N, O, Si, S, and Fe are present on the periphery of the Fe_3_O_4_–PVA@SH. We have calculated that the step-by-step method preparation of the drug carrier produces C : N : O : Si : S : Fe = 11.95 : 3.27 : 4.99 : 0.859 : 16.62 : 10.49, by contrast to the one-pot preparation method, which produces C : N : O : Si : S : Fe = 5.64 : 9.80 : 2.74 : 0.42 : 0.74 : 11.49. The spheroid is Fe_3_O_4_ and the outermost irregularity of the spheroid is a mixture of PVA and TIPTS. The uneven distribution of the individual elements of the EDS drug carrier prepared by the step-by-step method is caused by the Fe_3_O_4_ being encapsulated in PVA and greater exposure to C and O. Thus, it can be concluded that the C and O elements in the drug carrier synthesised by the one-pot method are more evenly distributed over the surface of the molecule than the product of the step-by-step method.

### 3.5 VSM analysis

**Fig. 5:**
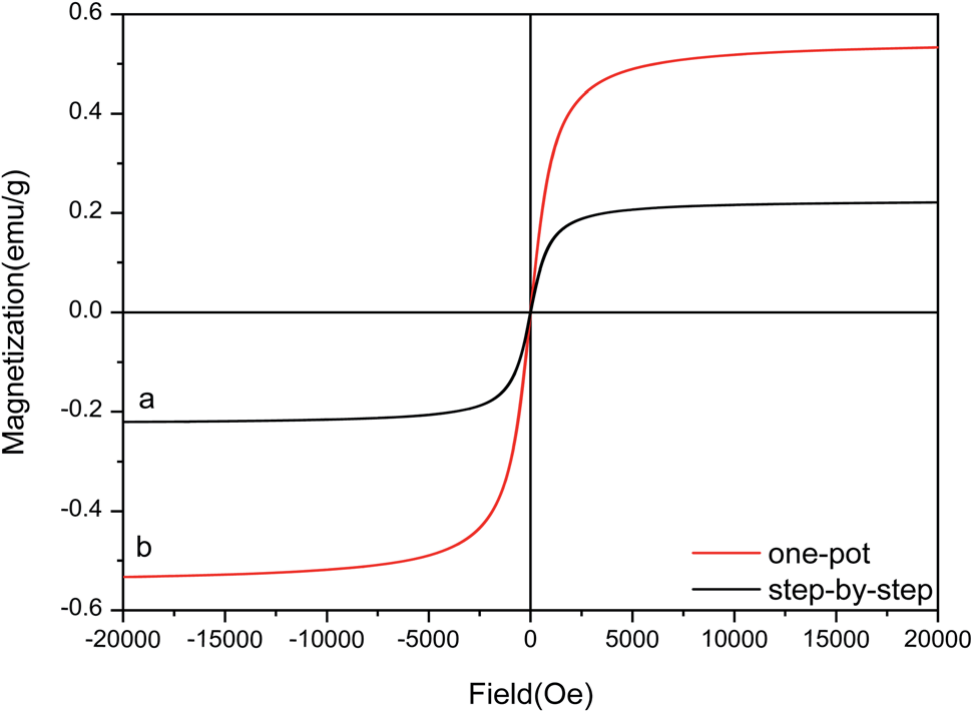
Hysteresis curves for Fe_3_O_4_–PVA@SH prepared using the step-by-step method (a) and the one-pot method (b).

The hysteresis curves for Fe_3_O_4_–PVA@SH prepared using the step-by-step method (a) and the one-pot method (b) can be seen in Fig. 5. The molecules from the one-pot method have a higher degree of magnetic saturation of the drug carrier than those of the step-by-step method. The difference in magnetic saturation is likely due to Fe_3_O_4_ being encapsulated by PVA during the first step of the step-by-step synthesis of Fe_3_O_4_–PVA@SH. Furthermore, as is shown in Fig. 4(a) and (b), the EDS analysis reveals that the content of Fe is higher from the one-pot method than from the step-by-step method. The increased presence of Fe could explain why the magnetic saturation of the product of the one-pot method is higher than that of the step-by-step method.

### 3.6 Contact angle and swelling rate analysis

As shown in Table 1, the contact angles of Fe_3_O_4_–PVA@SH were 59.32°, 61.87° and 54.38° with the step-by-step method and 68.21°, 69.32° and 66.75° with the one-pot method. Compared to the step-by-step method, the one-pot method results in a larger contact angle in the drug. We suspect the difference is due to the fact that during one-pot preparation, there is more time and space for TIPTS to be exposed to the surface of the carrier, resulting in a larger contact angle and increased fat solubility.

**Table 1:** Table of the contact angle and swelling rate of Fe_3_O_4_–PVA@SH prepared using the step-by-step method and one-pot method

Method	Contact angle (°)	Mean (°)	RSD (%)	Swelling rate (%)	Mean (%)	RSD (%)
Step-by-step	59.32	58.52	±3.81	148	148.33	±2.52
	61.87			151		
	54.38			146		
One-pot	68.21	68.09	±1.29	127	127.33	±4.51
	69.32			123		
	66.75			132		

The swelling rates of Fe_3_O_4_–PVA@SH prepared using the step-by-step method and one-pot method are shown in Table 1, with contact angles of 148, 151, and 146 for the step-by-step method and 127, 123, and 132 for the one-pot method. It can be seen that drug carriers synthesised *via* the step-by-step method show greater swelling compared with those synthesised using the one-pot method. This is likely caused by the high PVA content in the step-by-step method. We compared the C : O ratios in the drug carriers prepared using the DSC step-by-step method and the one-pot method, which were C : O = 11.95 : 4.99 and C : O = 5.64 : 2.74, respectively. This indicates that the step-by-step method produces more PVA than the one-pot method, and as result, the swelling ratio in the step-by-step synthesis is better than that of the one-pot method.

### 3.7 Drug carrying analysis

**Fig. 6:**
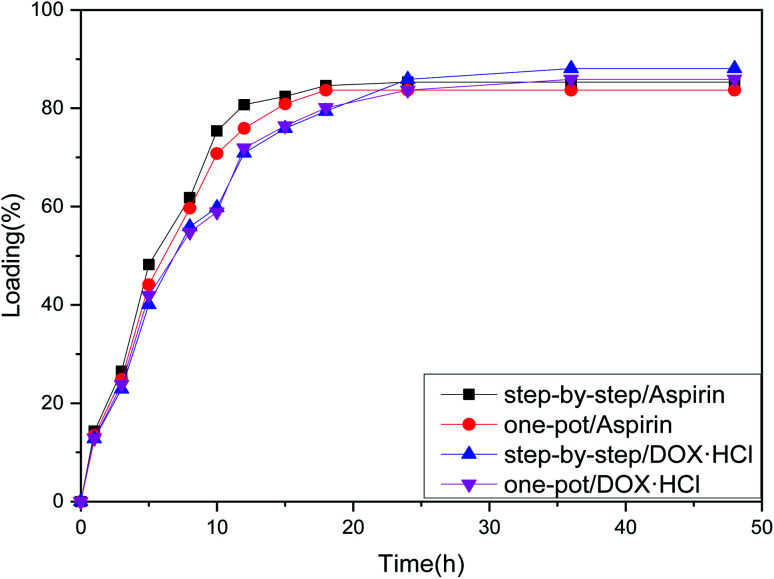
Loading folding line chart of Fe_3_O_4_–PVA@SH prepared using the step-by-step method and one-pot method.

From the loading folding line chart in Fig. 6 of Fe_3_O_4_–PVA@SH prepared using the two methods, it can be determined that there is a difference in the loading of Fe_3_O_4_–PVA@SH for aspirin and DOX·HCl. With the step-by-step preparation of the drug carrier for aspirin, loading was 85.3% ± 0.6, and with the step-by-step preparation of the drug carrier DOX·HCl, loading was 88.1% ± 0.9. With the one-pot preparation of the drug carrier for aspirin, loading was 83.7% ± 1.2, and with the one-pot preparation of the drug carrier DOX·HCl, loading was 85.2% ± 1.1. The drug carrier Fe_3_O_4_–PVA@SH had slightly lower loading of aspirin compared to DOX·HCl. This could be due to the fact that aspirin contains fewer polar functional groups per mole than DOX·HCl, which determines the load space available. The greater load seen with DOX·HCl was made possible by the sulfhydryl group (–SH), hydroxyl group (–OH), and carboxyl group (–COOH) on the drug carrier that form more hydrogen bonds with the polar functional group (–OH) of DOX·HCl. Despite the difference between the two drugs, the drug carrier Fe_3_O_4_–PVA@SH prepared using both methods demonstrates excellent loading properties, with the loading capacity of all drug carriers exceeding 70%.

### 3.8 Drug release analysis

**Fig. 7:**
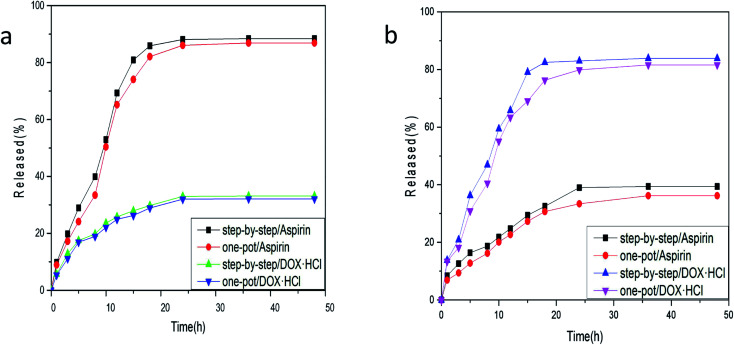
(a) Release folding line chart of Fe_3_O_4_–PVA@SH prepared using the step-by-step method and one-pot method at pH 7.2, 37 °C; (b) release folding line chart of Fe_3_O_4_–PVA@SH prepared using the step-by-step method and one-pot method at pH 4.7, 37 °C.

The release of aspirin from the drug carrier exceeds that of DOX·HCl by a significant margin, as can be seen from the release folding line chart in Fig. 7(a) of Fe_3_O_4_–PVA@SH at pH 7.2 and 37 °C prepared by the step-by-step method and one-pot method. The release of aspirin was 88.4% ± 1.1, with the step-by-step preparation, and 33.1% ± 0.6 for DOX·HCl at pH 7.2 and 37 °C. The release of aspirin was 86.9% ± 5.4 with the one-pot preparation, and 32.8% ± 0.8 for DOX·HCl. This is likely due to that pH 7.2 mimics the normal environment of body fluid, in which aspirin hydrolysis is reduced. However, such neutral conditions are not favourable to the binding of DOX·HCl with H^+^.

Although DOX·HCl release in Fig. 7(b) is higher at pH 4.7, this is the pH of simulated cell lysosomes. In an *in vitro* simulation at pH 4.7, 37 °C, the step-by-step preparation of drug carriers released 39.4% ± 0.3 of aspirin, and 83.9% ± 1.2 of adriamycin hydrochloride. The one-pot preparation of drug carriers released 36.2% ± 0.4 of aspirin, and 81.6% ± 1.6 of DOX·HCl. Aspirin is hydrolysed to *O*-hydroxybenzoic acid in an acidic environment, which results in a reduction of the aspirin content in the solution. However, the acidic environment facilitates the binding of DOX·HCl to H^+^, producing higher DOX·HCl content in the solution. In summary, there is more release of DOX·HCl in an acidic environment, whereas a neutral environment will result in more release of aspirin.

### 3.9 Comprehensive performance assessment

In order to evaluate the overall performance of the drug carriers prepared in this paper, we have referred to the drug carrier systems prepared by many teams for comparison. As shown in Table 2.

**Table 2:** Comparison of drug loading and drug release of different drug carrier systems

Sample	Loading	Released
mPVA gel^24^	70.92%	52.1%
C-PK/-SS-Hy-D NPs^27^	45.8%	88.6%
Asp@M-ZIF-8 (ref. 28)	300 cm g^−2^	100%
MP-PEG-FA NPs^29^	9.1%	75.0%
ZnO–DOX@ZIF-8 (ref. 30)	11.2%	Over 80%
One-pot Fe_3_O_4_–PVA@SH	85.3%	88.4%
Step-by-step Fe_3_O_4_–PVA@SH	88.1%	83.9%

## 4. Conclusion

We have successfully prepared a new magnetic targeting drug carrier Fe_3_O_4_–PVA@SH using the step-by-step method and the one-pot method. By loading aspirin and DOX·HCl, this study also demonstrated that the drug loading capacity of the Fe_3_O_4_–PVA@SH prepared using either of the two methods is greater than 70%. The level of drug release varies according to how the drug operates in different environments such that Fe_3_O_4_–PVA@SH can be developed into an effective magnetic targeting drug carrier. However, based on a combination of dissolution rate, particle size, EDS, drug loading and release properties Table 2 analysis, the performance of the drug carrier prepared by the stepwise method is superior. In consideration of production cost and the production process, by contrast, the simple one-pot method preparation is more efficient in terms of the industrial production of magnetic target drug materials. The low cost of the one-pot method of preparing drug carriers makes it possible to commercialise our research and offer more possibilities for the reduction and cure of the world's diseases. In summary, our study provides great opportunities in the continued use of magnetic carrier materials in various applications for magnetically targeted drug carriers.

## Author contributions

Conceived and designed the experiments: Zhen Shi and Yazhen Wang. Performed the experiments: Zhen Shi. Contributed reagents/materials/analysis tools: Yazhen Wang, Shaobo Dong and Tianyu Lan.

## Conflicts of interest

There are no conflicts to declare.

## Supplementary Material

RA-011-D1RA04256D-s001
