# Supplementary material for: Comparison of the performance of magnetic targeting drug carriers prepared using two synthesis methods
Source: RSC Adv. 2021 Jun 9;11(34):20670–8. doi: 10.1039/d1ra04256d (PMC9033997; doi:10.1039/d1ra04256d)
Supplement: RA-011-D1RA04256D-s001 [file RA-011-D1RA04256D-s001.pdf]

Electronic Supplementary Material (ESI) for RSC Advances.
This journal is © The Royal Society of Chemistry 2021

1 Preparation process of Fe_3O_4 nanoparticles：

Fe_3O_4 nanoparticles were prepared via ultrasonic precipitation. First, the ratio $FeCl_3 \cdot 6H_2O$:$FeCl_2 \cdot 4H_2O$ = 3:2 was dispersed into 200 mL of distilled water. Over the course of an hour, the whole system was sonicated. Then, 30 mL of ammonia (NH_3) was added to the solution and stirred at 65°C for 5 hours. Nitrogen was used to protect the entire reaction system. Finally, the product was rinsed repeatedly with water and then freeze-dried.

2. Particle size distribution of Fe_3O_4.

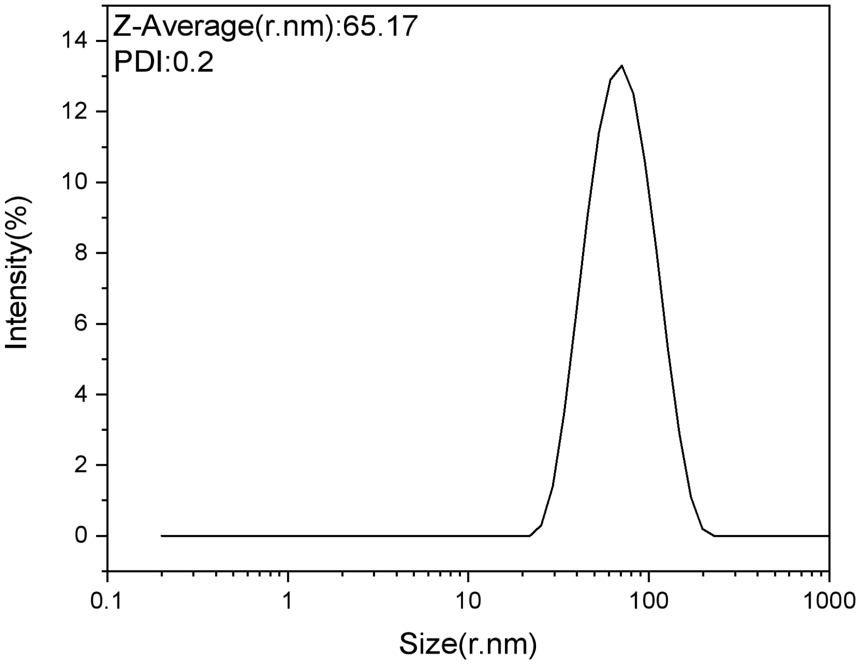

Figure 1 Particle size distribution of Fe_3O_4 nanoparticles

3 Information on the EDS elements table：

We use the quantity of matter equation to calculate the ratio of the amount of matter between each element：

$$n = \frac{m}{M}$$

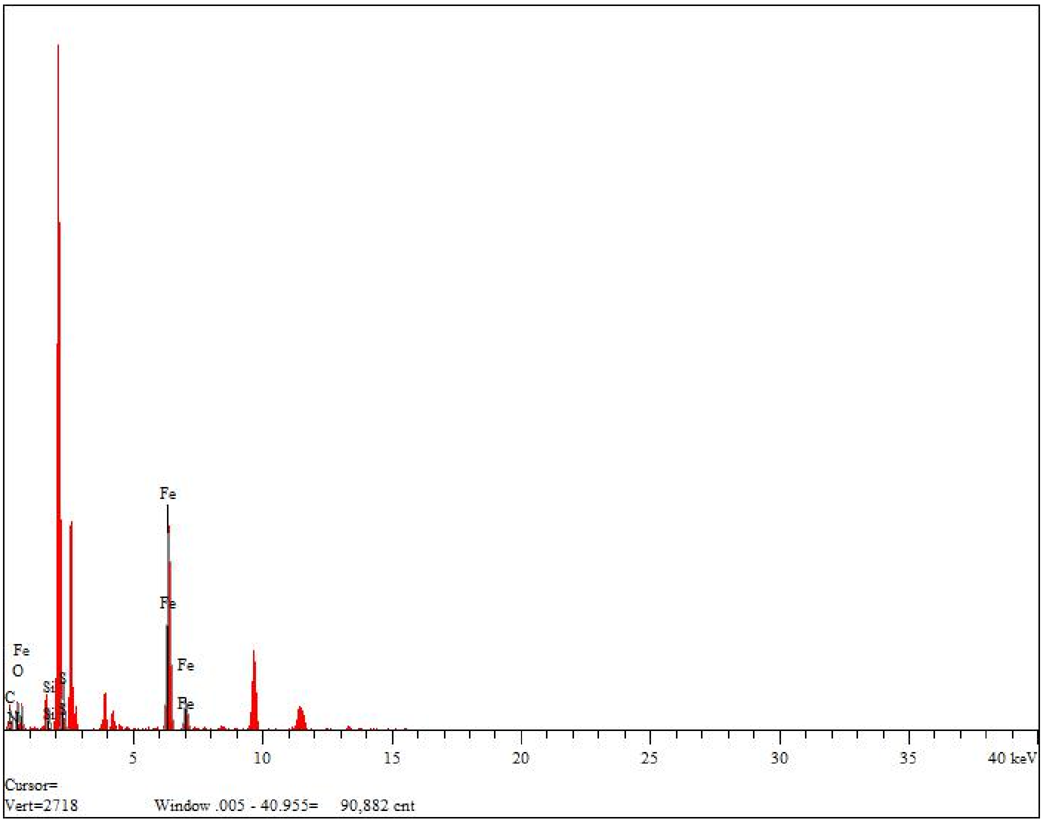

Figure 2 EDS for Fe_3O_4-PVA @SH prepared using the step-by-step method

Table 1 Elemental content of Fe_3O_4-PVA@SH prepared by step-by-step method

**Elt.**	**Line**	**Intensity (c/s)**	**Conc**	**Units**	**Error 2-sig**	**MDL 3-sig**	
C	Ka	16.90	14.337	wt.%	1.555	1.841	
N	Ka	0.48	0.457	wt.%	.465	.932	
O	Ka	21.87	7.986	wt.%	.721	.818	
Si	Ka	17.03	2.407	wt.%	.390	.541	
S	Ka	45.22	5.321	wt.%	.382	.474	
Fe	Ka	248.85	69.950	wt.%	1.389	.915	
			100.000	wt.%			Total

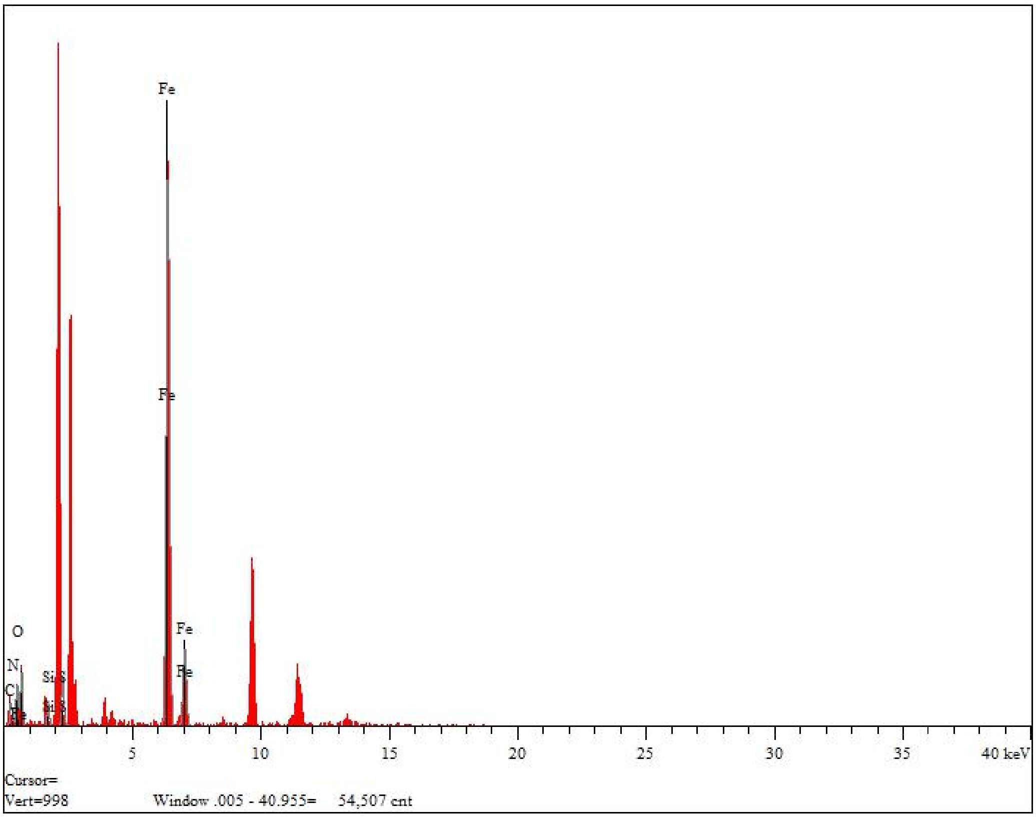

Figure 3 EDS for Fe_3O_4-PVA @SH prepared using the one-pot method

Table 2 Elemental content of Fe_3O_4-PVA@SH prepared by one-pot method

**Elt.**	**Line**	**Intensity (c/s)**	**Conc**	**Units**	**Error 2-sig**	**MDL 3-sig**	
C	Ka	7.21	6.778	wt.%	1.174	1.426	
N	Ka	1.44	1.372	wt.%	.825	1.161	
O	Ka	11.65	4.389	wt.%	.516	.560	
Si	Ka	6.60	1.183	wt.%	.293	.401	
S	Ka	16.54	2.373	wt.%	.299	.381	
Fe	Ka	253.63	83.905	wt.%	1.622	.979	
			100.000	wt.%			Total
